# Intravenous injection of cyclosporin A loaded lipid nanocapsules fights inflammation and immune system activation in a mouse model of diabetic retinopathy

**DOI:** 10.1007/s13346-023-01350-7

**Published:** 2023-05-19

**Authors:** Marilena Bohley, Andrea E. Dillinger, Barbara M. Braunger, Ernst R. Tamm, Achim Goepferich

**Affiliations:** 1 Department of Pharmaceutical Technology, University of Regensburg, Regensburg, 93053 Germany; 2 Institute of Pharmaceutical Sciences, Department of Chemistry and Applied Biosciences, ETH Zurich, Zurich, 8093 Switzerland; 3 Department of Human Anatomy and Embryology, University of Regensburg, Regensburg, 93053 Germany; 4 Institute of Anatomy and Cell Biology, Julius-Maximilians-University of Wuerzburg, Würzburg, 97070 Germany

**Keywords:** Diabetic retinopathy, Intravenous nanotherapy, RPE, Drug delivery, Lipid nanocapsules, Cyclosporin A

## Abstract

Inflammation and immune system activation are key pathologic events in the onset and escalation of diabetic retinopathy (DR). Both are driven by cytokines and complement originating from the retinal pigment epithelium (RPE). Despite the RPE’s pivotal role, there is no therapeutic tool to specifically interfere with the RPE-related pathomechanism. A therapy that addresses RPE cells and counteracts inflammation and immune response would be of paramount value for the early treatment of DR, where currently are no specific therapies available. Here, we utilized lipoprotein-mimetic lipid nanocapsules to deliver the anti-inflammatory and immunosuppressive drug cyclosporin A (CsA) to RPE cells. Using a mouse model of DR that mirrors all pathologic aspects of human DR, we demonstrate that intravenously applied CsA-loaded lipid nanocapsules comprehensively counteract inflammation and immune system activation. One single injection suppressed the expression of pro-inflammatory cytokines, dampened macrophage infiltration, and prevented macrophage and microglia activation in eyes with DR. This work shows that CsA-loaded lipid nanocapsules can offer new avenues for the treatment of DR.

**Graphical Abstract:**

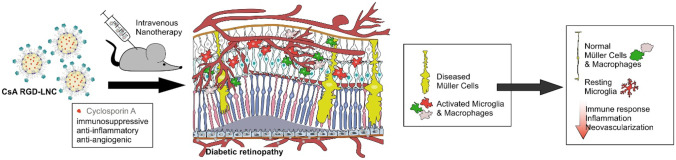

## Introduction

DR is a progressive and multifactorial retinal disease that is one of the leading causes of visual impairment and blindness [1]. It is the most frequently occurring complication of diabetes mellitus. As the worldwide prevalence of diabetes mellitus is predicted to reach more than 700 million by 2045, the prevalence of DR is also expected to increase dramatically [2]. DR affects most patients with diabetes mellitus as it is initiated by damage to the retina due to increased retinal blood sugar levels [3]. Clinically, early (non-proliferative) stages of DR are characterized by microaneurysms, hard exudates, and hemorrhages causing no or only moderate visual impairment. Over time, early DR progresses into proliferative DR. Growth factor dysregulations due to ischemic and inflammatory conditions subsequently induce endothelial cell proliferation and retinal neovascularization [4]. The newly formed vessels are leaky and cause diabetic macular edema and/or severe hemorrhages, leading to retinal detachment and destruction of the neural retina. This causes severe visual impairment and blindness [4].

Even though the precise underlying mechanism inducing retinal cell degeneration and damage are not well understood, inflammation and exuberant immune system activation are detrimental factors and may represent the inciting factors leading to the complex pathology [5, 6]. In recent years, the RPE, a cell monolayer located between the choriocapillaris and photoreceptors, was recognized as cellular key player in the pathogenesis of DR [7]. RPE cells produce and secrete crucial growth factors, such as the pro-angiogenic vascular endothelial cell growth factor (VEGF) and its counterpart, the transforming growth factor β (TGF-β). Additionally, they regulate inflammation and immune responses via cytokine and complement expression [8]. To date, it is also known that RPE cells regulate and control the unique defense system of the eye, consisting of retinal immune cells, including microglia and macrophages, and the complement system. By being able to control retinal neovascularization, inflammation, and activation of immune cells, RPE cells play a pivotal role in the pathogenesis and progression of DR [7, 9].

However, currently, there are no treatment options available to address these cells [10]. More so, there is an unmet need for therapeutic approaches addressing early disease states and preventing disease progression to severe proliferative DR [11]. We believe that targeted drug delivery to RPE cells with the aim to comprehensively fight inflammation, immune system activation, and neovascularization can have significant implications for the treatment of DR and might further prevent progression from the non-proliferative to the proliferative stage.

In principle, drug delivery to the RPE can be achieved by two different approaches: either using intravitreal injections or systemic administration. After intravitreal administration, the drug delivery system must diffuse through the whole vitreous, escape phagocytosis by Müller cells, and most importantly, distinguish between various retinal cells, including the highly sensitive photoreceptors and the target cells. Even if trafficking through multiple barriers—on the way from the vitreous to the RPE—can be achieved, drug carriers often lack cell specificity for RPE cells and are therefore prone to initiate inflammatory responses and trigger serious off-target effects. In contrast, upon reaching the systemic circulation, the drug carrier must only extravasate from the choriocapillaris and diffuse through the Bruch’s membrane, an extracellular matrix, to reach the RPE [7].

In this work, we utilized cyclo(-Arg-Gly-Asp-D-Phe-Cys) (cRGD)-modified lipoprotein-mimetic lipid nanocapsules (RGD-LNCs) to target RPE cells after intravenous injection. As previously reported, RGD-LNCs extravasate from the choroidal vessels and accumulate in RPE cells upon intravenous administration. To address the multifactorial pathomechanism, we loaded them with immunosuppressive, anti-inflammatory, and anti-angiogenic CsA [12]. Oral administration of CsA to transplantation patients suffering from DR has been shown to alleviate disease progression significantly but moderately [13–15]. In doing so, CsA has demonstrated its enormous, yet unexploited, therapeutic potential but suffered from insufficient availability in the eye [16].

We hypothesized that delivery of CsA to RPE cells using CsA-loaded RGD-LNCs (CsA RGD-LNCs) has the potential to synergistically counteract immune responses, inflammation, and neovascularization of DR. The goal of this study was to investigate if a single intravenous dose of CsA RGD-LNCs can dampen inflammation and immune response by suppressing pro-inflammatory cytokine expression and preventing microglia/macrophage activation. We used a TGF-β related mouse model of DR that mimics all relevant pathologic aspects of human DR. We first characterized inflammatory and immune events in TGF-β receptor type 2 (TGFβ-R2) deficient mice (DR mice), focusing on cytokine expression, Müller cell reactivity, and immune cell activation. Thereafter, we determined the therapeutic potential of intravenously applied CsA RGD-LNCs compared to free CsA solution and a PBS-treated control by assessing the effects on cytokine expression levels, Müller cell reactivity, and microglia/macrophage activation.

## Results and discussion

### TGFβ-R2 deletion in mice causes pathological characteristics of human DR

**Fig. 1:**
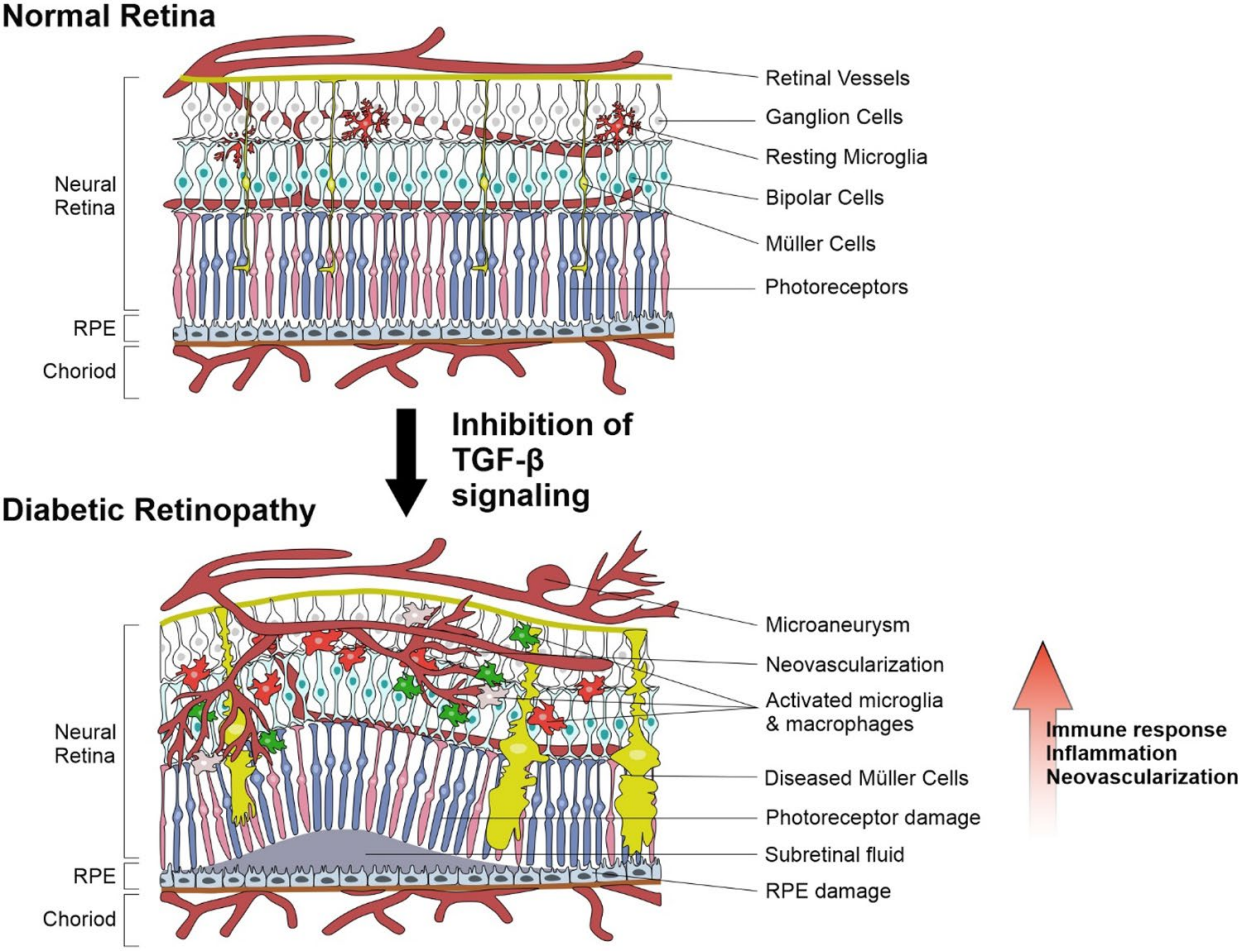
Inhibition of TGF-β signaling results in essential characteristics of DR. (1) Formation of abundant vascular microaneurysms, newly formed and leaky vessels; (2) invasion of activated and reactive microglia cells and macrophages; (3) activation of Müller cells; (4) neuronal and photoreceptor cell degeneration; (5) retinal detachment, breakdown of the blood-retinal barrier, retinal, and vitreal hemorrhages. Overall, resulting in exuberant immune system activation, inflammation, and pronounced neovascularization

Constitutive TGF-β signaling is detrimentally important for maintaining the immune privilege of the eye, retinal homeostasis, and proper function of the retinal vasculature [17]. Inhibiting TGF-β signaling results in apoptosis of vascular cells, formation of leaky capillaries, activation, migration, and accumulation of microglia and macrophages (Fig. 1) [18, 19]. Consequently, pro-inflammatory cytokines, chemokines, complement, pro-angiogenic factors, and activated macrophages/microglia are driving the progression from early to proliferative DR [20].

Recently, it was shown that the impairment of ocular TGF-β signaling in mice causes structural changes that largely mimic human DR. It was shown that the lack of TGF-β signaling induces typical vascular characteristics of proliferative DR regarding structural changes of retinal capillaries, including the formation of abundant microaneurysms, leaky capillaries, and retinal hemorrhages [18, 19].

**Fig. 2:**
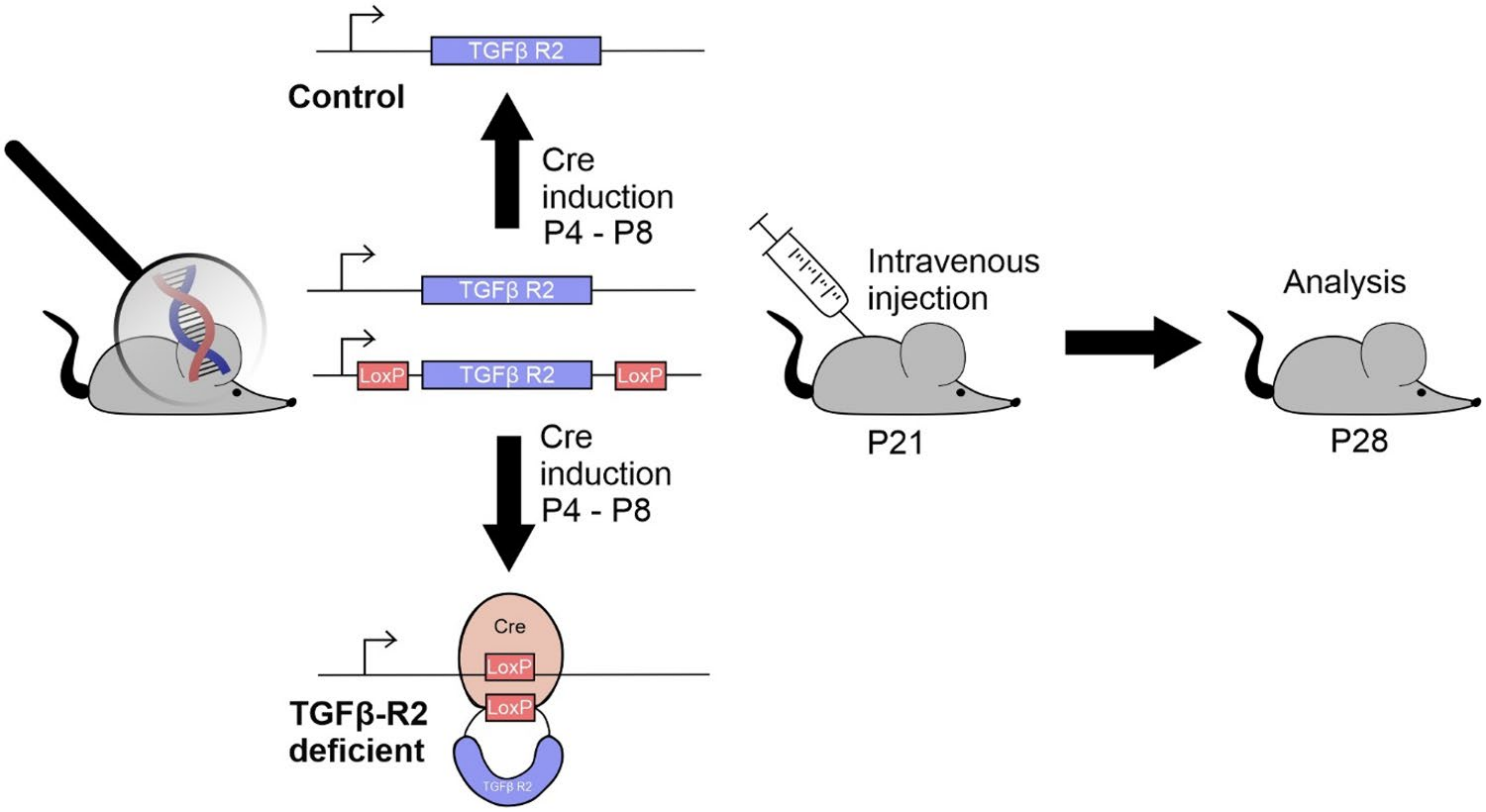
Scheme depicting the generation of TGFβ-R2 deficient and control mice and the subsequent intervention regime. P: postnatal day. To induce the tamoxifen-dependent Cre recombinase and delete TGFβ-R2 in the ocular tissue, mice were treated from P4 to P8 with tamoxifen eye drops. After TGFβ-R2 deletion, TGFβ-R2 deficient (referred to as DR mice) and control mice were treated at P21, receiving one intravenous injection of either PBS, CsA solution (referred to as free CsA), or CsA RGD-LNCs, and were analyzed at P28

TGF-β signaling was impaired via conditional deletion of the essential TGFβ-R2 in the ocular tissue of mice. This was accomplished by inducing a tamoxifen-dependent Cre recombinase, which, after local tamoxifen treatment, subsequently suppressed TGFβ-R2 expression in the mouse eye. The resulting TGFβ-R2 flox/flox CAGGCre-ER mice (= TGFβ-R2 deficient mice; referred to as DR mice) were used as experimental mice, whereas the littermates with two unrecombined TGFβ-R2 flox/flox alleles were used as healthy control (Fig. 2).

**Fig. 3:**
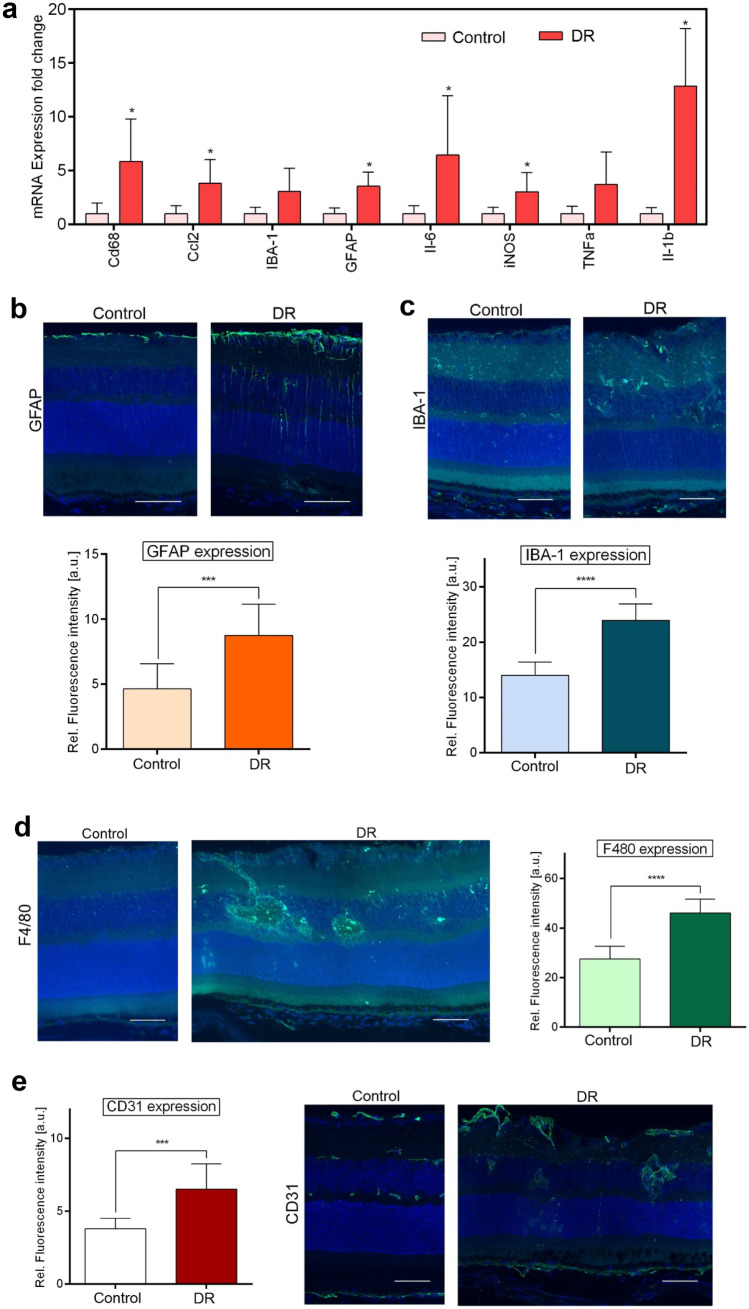
Assessment of inflammation and immune system activation of DR mice. **a** Quantitative analysis of mRNA expression levels in retinal tissues using RT-qPCR. The results are normalized to healthy controls and represented as mean ± SD. **b** Imaging of GFAP expression in the posterior eye. Blue: DAPI staining of cell nuclei; green: GFAP. Quantitative analysis of GFAP fluorescence density from cryosections. **c** Imaging of IBA-1 expression in the posterior eye. Blue: DAPI staining of cell nuclei; green: IBA-1. Quantitative analysis of IBA-1 fluorescence density from cryosections. **d** Imaging of F4/80 expressions in the posterior eye. Blue: DAPI staining of cell nuclei; green: F4/80. Quantitative analysis of F4/80 fluorescence density from cryosections. **e** Imaging of CD31 expression in the posterior eye. Blue: DAPI staining of cell nuclei; green: CD31. Quantitative analysis of CD31 fluorescence density from cryosections. Scale bars: 50 μm. Results are presented as mean ± SD of at least *n* = 5 mice per treatment group. Levels of statistical significance are indicated as * *P* ≤ 0.05, ** *P* ≤ 0.01, *** *P* ≤ 0.001, and **** *P* ≤ 0.0001. *P*-values determined by unpaired Student’s *t*-test

To assess whether we can use this model to test the effects of CsA RGD-LNCs on inflammation and immune response, we first examined and quantified inflammatory and immune events in the eyes of DR mice. The immunovascular axis, the cross-talk of RPE cells with microglia, macrophages, and vascular endothelial cells, is the key mediator of inflammation and immune system activation. Thus, we first analyzed mRNA expression levels of pro-inflammatory cytokines and markers for the reactivity of glia cells. Glia cells are resident immune cells that play important roles in maintaining tissue homeostasis. However, inappropriate microglia activation due to pathogenesis drives inflammation-mediated retinal cell degeneration and is a major contributor to inflammatory and immune processes underlying early DR [21, 22]. To assess glia cell activation, we analyzed the mRNA expression profile of microglia activation markers (CD68), chemokines (Ccl2), and markers of gliosis (glial fibrillary acidic protein (GFAP)) (Fig. 3a). In contrast to control mice, DR mice showed significant increases in CD68, Ccl2, and GFAP. Additionally, as activated retinal glia cells release NO, which induces neuronal cell degeneration, levels of cytokine-inducible nitric oxide synthase (iNOS) were assessed. DR mice revealed significantly elevated iNOS levels.

We assessed the increase of pro-inflammatory cytokines as another important marker for inflammatory and immune events. Mainly expressed by Müller and RPE cells, the pro-inflammatory interleukins (ILs) IL-1β and IL-6, are the most potent. Both stimulate, activate, and recruit immune cells, driving immune and inflammatory responses [23]. The dramatic increase in these pro-inflammatory cytokines in the retina of DR mice additionally confirms inflammation and immune system activation (Fig. 3a). Taken together, the analysis of pro-inflammatory mediators and markers for immune cell activation strongly indicated inflammatory and immune system-mediated processes in the eye of DR mice similar to the events in human early DR.

Next, we determined the extent of glia cell activation. To that end, we first used immunohistochemistry against GFAP to confirm increased Müller cell gliosis on the protein level (Fig. 3b). In control mice, immunohistochemical GFAP labeling was restricted to cells on the inner retinal surface, indicating normal presence of GFAP in astrocytes and/or Müller cell end-feet. In DR mice, radial GFAP expression was seen throughout the retina, revealing highly reactive Müller cells and the typical gliosis reaction of Müller cells due to inflammation and/or damage of the retina. Quantitative analysis confirmed these observations and revealed significantly higher GFAP protein levels in DR mice compared to control mice.

After determining the reactivity of Müller cells, we used an antibody against IBA-1 to visualize macrophages and microglia. In control eyes, IBA-1 positive cells were detected at their typical localization, including retinal surfaces, inner and outer plexiform layers, and they also exhibited the shape of resting microglia (Fig. 3c). Whereas, in the retina of DR mice, IBA-1-positive cells migrated to retinal vessels and started to form a layer around them. They also changed their shape from that resting microglia to reactive microglia. These observations were supported by significantly increased levels of IBA-1 in the retina of DR mice.

To investigate the extent of activation and localize activated microglia and macrophages, immunohistochemistry for F4/80 was used. In control eyes, F4/80 positive cells were absent, whereas in DR eyes, high amounts of activated immune cells were found migrating to or in near proximity of diseased retinal vessels (Fig. 3d). F4/80 levels in the retina of DR mice are significantly increased compared to control mice, reflecting the accumulation of activated microglia and macrophages.

After the assessment of cellular changes due to inflammation and immune responses, we determined the extent of retinal neovascularization. To that end, retinas of DR and control mice were stained for CD31, an endothelial cell marker staining choroidal and retinal vessels. Figure 3e reveals that retinas of DR mice showed enlarged retinal vessels and various areas of retinal neovascularization compared to control. These retinal vessels were tortuous and appeared blurry, indicating vascular leakage and hemorrhage. The proliferation of endothelial cells is further demonstrated by an increase in CD31 levels in the retina of DR mice.

Overall, we showed that the impairment of ocular TGF-β signaling via conditional deletion of TGFβ-R2 does not only affect the retinal vasculature, leading to events mimicking proliferative DR but also induce inflammation and immune response, events driving the onset and progression of DR. By perfectly reflecting the multifactorial pathogenesis of both early and late-stage DR, TGFβ-R2 deficient mice provides a suitable in vivo model to test our nanotherapeutic.

### Systemic CsA RGD-LNCs counteract inflammation and immune system activation of DR

**Fig. 4:**
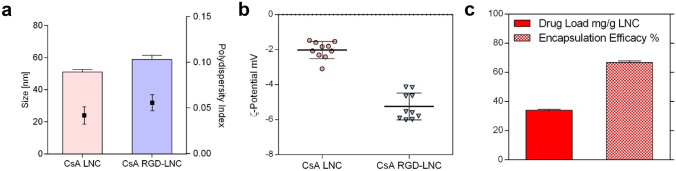
Characterization of CsA RGD-LNCs. **a** Size and polydispersity index of CsA RGD-LNC. **b** ς-potential of CsA RGD-LNC. **c** Absolute drug load in mg CsA per g LNC dispersion and percentage encapsulation efficacy (experimental drug payload/theoretical drug payload). Results are presented as mean ± SD of *n* = 10 (**a** and **b**) and *n* = 3 (**c**) independent experiments

After the successful demonstration that DR mice exhibit inflammatory and immune events like those underlying human DR, we assessed if a single intravenous injection of CsA RGD-LNCs can attenuate inflammation and immune system activation. We treated DR and control mice with a single intravenous injection of either PBS, CsA RGD-LNCs, or a CsA solution (free CsA) with the same concentration of CsA (0.68 mg/ml). CsA RGD-LNCs exhibited a size of approximately 60 nm, slightly negative zeta potential, and a drug load of 34 mg CsA/g LNC (Fig. 4).

**Fig. 5:**
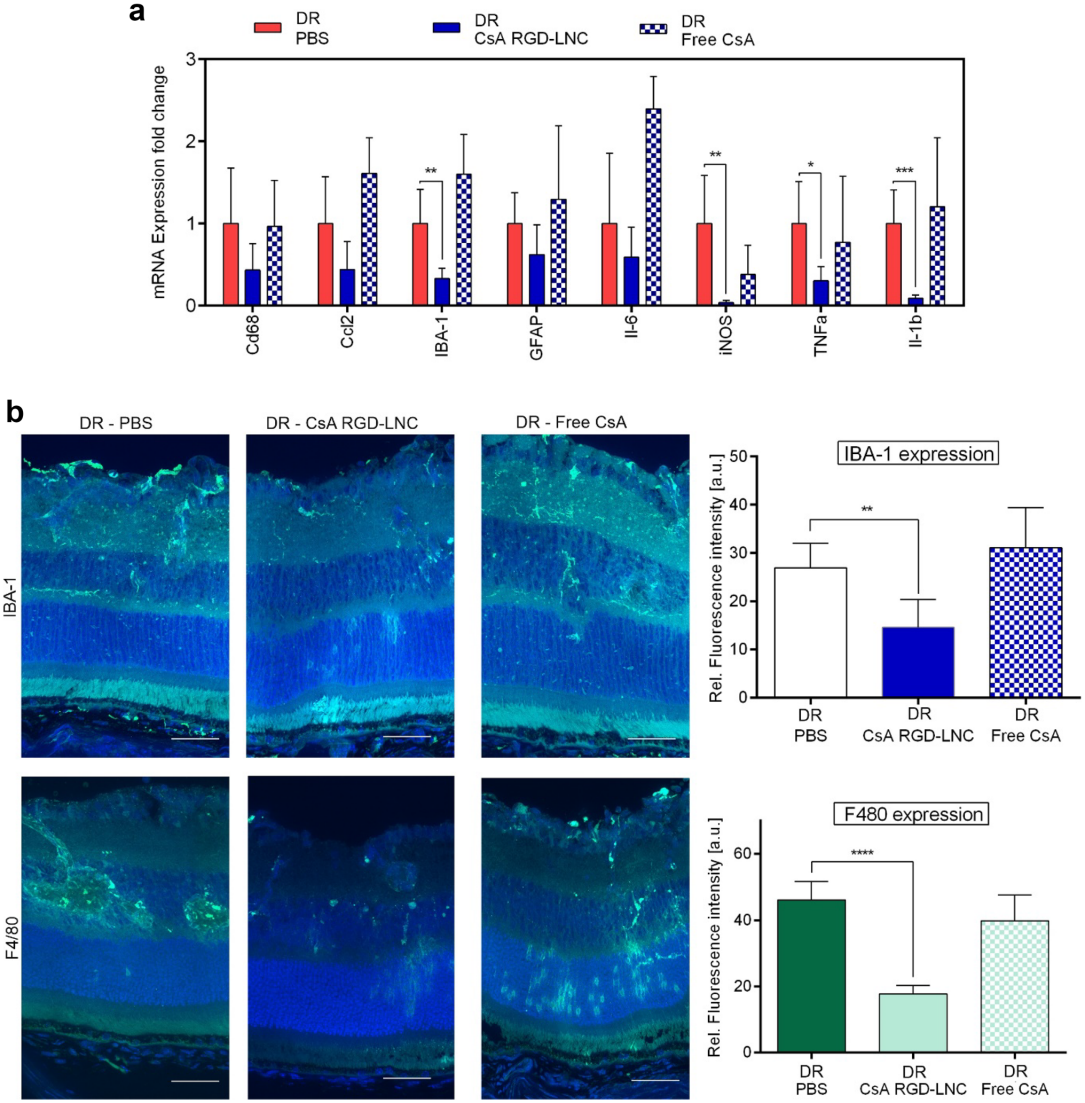
Assessment of the attenuation of inflammation and immune system activation after the systemic treatment of DR mice. **a** Quantitative analysis of mRNA expression levels in retinal tissues using RT-qPCR. The results are normalized to DR mice treated with PBS. **b** Imaging of IBA-1 and F4/80 expression in the posterior eye. Blue: DAPI staining of cell nuclei; green: IBA-1; F4/80. Quantitative analysis of IBA-1 or F4/80 fluorescence density from cryosections immunohistochemically stained with antibodies. Scale bars: 50 μm. Results are presented as mean ± SD of at least *n* = 5 mice per treatment group. Levels of statistical significance are indicated as * *P* ≤ 0.05, ** *P* ≤ 0.01, *** *P* ≤ 0.001, and **** *P* ≤ 0.0001. *P*-values determined by one-way ANOVA

To evaluate the effects of CsA RGD-LNC treatment on immune system activation and inflammation mice were analyzed at P28. First, we assessed the expression of pro-inflammatory cytokines, microglial, and macrophage activation markers using RT-qPCR (Fig. 5a).

Compared to free CsA, intravenous CsA RGD-LNC therapy significantly reduced exaggerated levels of IBA-1 and iNOS, implying reduced microglia cell reactivity. In addition to microglia markers, pro-inflammatory cytokines IL-1β and tumor necrosis factor-alpha (TNF-α) expression were significantly suppressed by the CsA RGD-LNC treatment compared to the PBS and free CsA treatment groups. While IL-1β directly affects immune cells, TNF-α affects many retinal cell types, including RPE cells, endothelial cells, and macrophages, as it is known to stimulate IL expression and exhibit proinflammatory, angiogenic, and fibrogenic potential. More so, the suppression of TNF-α using daily applied non-steroidal anti-inflammatory drugs has been shown to reduce inflammation, diabetic blood-retinal barrier breakdown, endothelial cell degeneration, and death [24, 25]. Thus, significant reduction of IBA-1, iNOS, IL-1β, and TNF-α expression after CsA RGD-LNC treatment in retinal tissues may lead to attenuated inflammation, microglia, and macrophage activation and inhibit DR disease progression.

To further investigate the anti-inflammatory and immuno-suppressive activity of CsA RGD-LNC therapy, retinal macrophage and microglia reactivity was determined using immunohistochemistry (Fig. 5b). While retinas of PBS and free CsA-treated DR mice displayed large amounts of IBA-1 positive cells in their reactive shape, the images of CsA RGD-LNC treated DR mice demonstrated a reduction of activated immune cells and revealed their shape-shift into the resting/normal state. The significant reduction of IBA-1-positive cells due to CsA RGD-LNC therapy additionally proves the successful reduction of microglia and macrophage accumulation in the diseased retina. Besides the reduction of microglia and macrophage presence, the effects of CsA-RGD-LNC treatment on the degree of activation and the localization of microglia and macrophages were investigated. CsA-RGD-LNC-treated mice demonstrated a significant reduction of reactive immune cells. In contrast, free CsA alone had no effects either on macrophage or microglia activation and accumulation, indicating that the drug delivery system is essential.

**Fig. 6:**
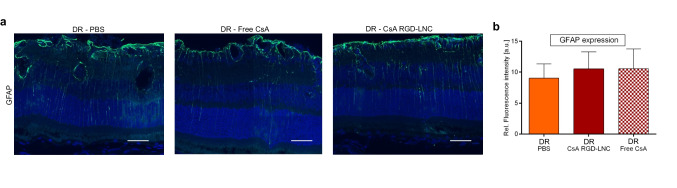
Assessment of Müller cell activation after systemic treatment of DR mice. **a** Imaging of GFAP expression in the posterior eye. Blue: DAPI staining of cell nuclei; green: GFAP. Scale bars: 50 μm. **b** Quantitative analysis of GFAP fluorescence density from cryosections immunohistochemically stained with GFAP antibody. Results are presented as mean ± SD of at least *n* = 5 mice per treatment group. Levels of statistical significance are indicated as * *P* ≤ 0.05, ** *P* ≤ 0.01, *** *P* ≤ 0.001, and **** *P* ≤ 0.0001. *P*-values determined by one-way ANOVA

Even though CsA RGD-LNC treatment revealed no significant effects on reactive Müller cells (Fig. 6), one single injection successfully counteracted both pro-inflammatory cytokine expression and immune cell activation in mice with DR. Taken together, CsA RGD-LNCs successfully attenuated inflammation and immune responses.

Finally, as CsA can suppress the intracellular VEGF signaling pathway and has further been shown to alleviate endothelial cell sprouting and proliferation in vitro and in vivo [12, 26, 27], the effect on neovascularization was assessed.

**Fig. 7:**
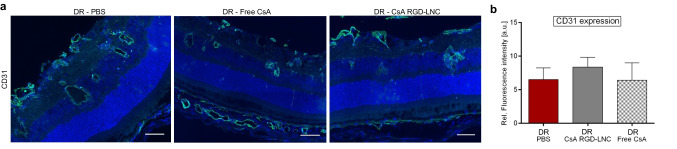
Assessment of endothelial cell proliferation after systemic treatment of DR mice. **a** Imaging of CD31 expression in the posterior eye. Blue: DAPI staining of cell nuclei; green: CD31. Scale bars: 50 μm. **b** Quantitative analysis of CD31 fluorescence density from cryosections immunohistochemically stained with CD31 antibody. Results are presented as mean ± SD of at least *n* = 5 mice per treatment group. Levels of statistical significance are indicated as * *P* ≤ 0.05, ** *P* ≤ 0.01, *** *P* ≤ 0.001, and **** *P* ≤ 0.0001. *P*-values determined by one-way ANOVA

Even though microscopy pictures suggest that CsA RGD-LNCs decrease the extent of neovascularization and the enlargement and number of retinal vessels compared to the PBS-treated control, no significant difference can be determined using quantitative fluorescence density measurements of CD31 (Fig. 7). This lack of efficacy regarding endothelial cell proliferation could be explained by two facts: First, one single intravenous injection of CsA RGD-LNCs on P21 could not be enough to successfully counteract endothelial cell proliferation induced by TGF-β signaling impairment. And second, the antiproliferative effect of CsA relies in part on suppressing the TGF-β-related increase of VEGF production in RPE cells, restoring the balance between the counterparts TGF-β and VEGF [28]. However, as our disease model is based on the impairment of TGF-β signaling and aims to fully eradicate TGF-β effects by deletion of TGFβ-R2, it is impossible to restore this balance. To specifically investigate the effects of intravenous CsA RGD-LNCs on neovascularization of proliferative DR, a TGF-β independent disease model would be needed.

Nevertheless, we demonstrated that intravenously applied CsA RGD-LNCs attenuate pro-inflammatory cytokine expression, macrophage and microglia accumulation, activation, and migration. One single intravenous injection was enough to successfully suppress inflammation and immune system activation for at least 1 week. This is highly beneficial, as pronounced microglia and macrophage activation is known to further trigger the inflammatory and angiogenesis cascade [20]. As CsA additionally bears anti-angiogenic potential by inhibiting intracellular VEGF signaling and counteracting TGF-β driven VEGF expression of RPE cells, systemic CsA RGD-LNCs could also be advantageous for the treatment of neovascular events of proliferative DR [29]. As CsA, in contrast to traditional intravitreal anti-VEGF therapies, addresses the intracellular VEGF signaling and production, the nanotherapeutic may be complementary to current standard therapies [30, 31]. Furthermore, intravenous nanotherapy can improve compliance and safety, as intravitreal therapies pose a significant financial burden as well as patient discomfort and their invasive nature bears the risks of intraocular bleedings, infections, retinal detachment, and most importantly lack of retinal cell specificity [32, 33].

## Conclusion

Ocular TGFβ-R2 deletion in mice induces retinal inflammation, immune system activation, and neovascularization, precisely mimicking the pathology of human DR. We used intravenously applied CsA RGD-LNCs to deliver the highly potent immunosuppressive, anti-inflammatory, and anti-angiogenic CsA to RPE cells. CsA RGD-LNCs comprehensively counteract inflammation and immune system activation. It was shown that a single intravenous injection of CsA RGD-LNC efficiently dampens pro-inflammatory interleukin and TNF-α expression. The treatment with CsA RGD-LNC attenuated inflammatory and immune responses by suppressing macrophage and microglia migration, infiltration, and activation. The CsA RGD-LNC treatment induced a phenotype change in macrophages and microglia from activated to resting state. This can break the inflammatory cycle, resulting in decreased microglia activation, macrophage recruitment and dampened pro-inflammatory cytokine release. The intravenous nanotherapy has been shown to be beneficial in terms of RPE cell specificity and drug delivery efficacy and may provide new therapeutic opportunities for the treatment of other ocular diseases with RPE involvement, such as age-related macular degeneration and retinopathy of prematurity. This study shows that targeted delivery of CsA to RPE cells and appropriately “manipulating” the immunovascular axis can have profound impacts on pathological events driving DR progression.

## Materials and methods

### Materials

Cyclosporin A (CsA) was obtained from Pharma Stulln GmbH (Stulln, Germany). Kolliphor^®^ HS15 was obtained from BASF. Lipoid^®^ S75-3 was obtained from Lipoid GmbH (Ludwigshafen, Germany). Miglyol^®^ 812 (MCT) was purchased from Caesar & Loretz GmbH (Hilden, Germany). 1,1′-Dioctadecyl-3,3,3′,3′-tetramethylindocarbocyanine perchlorate (DiI) was purchased from Invitrogen (Thermo Fisher Scientific, Waltham, MA, USA). 1,2-Distearoyl-sn-glycero-3-phosphoethanolamine-N[maleimide(polyethyleneglycol)-2000] (ammonium salt) (DSPE-PEG2000-maleimide) was purchased from Avanti Polar Lipids Inc. (Alabaster, AL, USA) and cyclo(-Arg-Gly-Asp-D-Phe-Cys) acetate salt (RGD) from Bachem Distribution Service GmbH (Weil a. Rhein, Germany). Dulbecco’s phosphate-buffered saline (PBS) was acquired from Gibco^®^ Life Technologies (Waltham, USA). Purified water was obtained from a Milli-Q System from Millipore (Schwalbach, Germany). All other materials and reagents in analytical grade were obtained from Merck (Taufkirchen, Germany).

### Lipid nanocapsule preparation and characterization

LNC were prepared as previously described [12]. In short, 887.5 mg Kolliphor^®^ HS15 40% (*w*/*w*), 30 mg Lipoid^®^ S75-3, 415 mg 8.5% (*w*/*w*) CsA-MCT solution, 1.6 mg 1.5% (*w*/*w*) DiI, 12 mg NaCl, and 655.8 mg water were heated in a phase inversion process of 3 cycles between 90 and 60 °C. In the third cycle, water was added at the phase inversion temperature, leading to stable LNC formation. The final dispersion was filtered through a 0.22 µm regenerated cellulose (RC) membrane for sterilization and stored at room temperature in the dark until further use. For cRGD peptide grafting, cRGD was first coupled to DSPE-PEG2000-maleimide (2 h, RT, 500 rpm). Next, the conjugates were inserted in the shell of the LNC by using the post-insertion method as previously described (3 h, 37 °C, 500 rpm) [12]. For purification and concentration, the modified RGD-LNCs were dialyzed against DPBS overnight using Spectra/Por^®^ Float-A-Lyzer^®^ G2 MWCO 300 kDa and subsequently centrifuged twice (15 min, RT, 4000 g) using a 100 kDa molecular weight cutoff Amicon^®^ Ultra-4 centrifugal device.

Size and ξ‐potential of the resulting particles were measured in 10% DPBS (*v*/*v*) at a constant temperature of 25 °C with a ZetaSizer Nano ZS (Malvern Instruments).

### Mice

Mice with two floxed alleles of TGFβ-R2 (TGFβ-R2 fl/fl) were crossed with heterozygous CAGGCre-ER mice, resulting in TGFβ-R2 fl/fl;CAGGCre-ER, referred to as TGFβ-R2 deficient mice. TGFβ-R2 fl/fl littermates with two unrecombined TGFβ-R2 alleles served as control mice and are referred to as controls. Genetic backgrounds were 129SV and CD1. All mice were reared in a light/dark cycle of 12 h (lights on at 7 am). Genotypes were identified by isolating genomic DNA from ear biopsy specimens and tested for transgenic Cre sequences and floxed TGFβ-R2 sequences using PCR. For Cre-PCR analysis, primers were 50-ATGCTTCTGTCCGTTTGCCG-30 (sense) and 50-CCTGTTTTGCACGTTCACCG-30 (antisense). The thermal cycle profile was denaturation at 95 °C for 30 s, annealing at 61 °C for 30 s, and 30-s extension at 72 °C for 35 cycles. For genotyping of TGFβ-R2 fl/fl mice, primers were 50-GCAGGCATCAGGACCCAGTTTGAT-CC-30 (sense) and 50-AGAGTGAAGCCGTGGTAGGT-GAGCTTG-30 (antisense). The thermal cycle profile was denaturation at 95 °C for 30 s, annealing at 62 °C for 30 s, and 35-s extension at 72 °C for 35 cycles.

### Induction of conditional TGFβ-R2 deletion

To activate Cre recombinase in TGFβ-R2 deficient mice, the conditional knockout animals and their respective control littermates were equally treated with tamoxifen-containing eye drops from postnatal day (P) 4 to P8, as described previously [18]. Tamoxifen was diluted in corn oil to a final concentration of 5 mg/ml. From P4 to P8, eye drops at a volume of 10 ml were pipetted onto the closed eyelids of mouse pups three times a day.

### Treatment of mice

After the induction of TGFβ-R2 deletion from P4 to P8, TGFβ-R2 deficient (referred to as DR mice) and control mice were treated at P21 with either 30 µl CsA RGD-LNCs (10 mg/ml), CsA solution (Sandimmun 50 mg/ml, Novartis, Basel, Switzerland) diluted with isotonic saline solution to a final concentration of 0.68 mg/ml, or DPBS by injection via the vena jugularis after anesthesia with ketamine (125 mg per kg body weight) and xylazine (80 mg per kg body weight). In total, 7 days after the treatment, at P28, mice were anesthetized with ketamine (125 mg per kg body weight) and xylazine (80 mg per kg body weight), killed through perfusion fixation with 4% paraformaldehyde (PFA), and eyes were enucleated and processed as described below.

### Immunohistochemistry

For immunohistochemical analysis, eyes were fixed for 4 h in 4% PFA, washed extensively in phosphate buffer (0.1 M, pH 7.4) (PB), incubated in 10%, 20%, and 30% sucrose/PB solution for 12 h each at 4 °C. Cryoprotected eyes were embedded in Tissue Tek O.C.T. Compound (Sakura Finetek) and subsequently frozen in liquid nitrogen. Finally, they were cut into 12 µm sections using an HM 500 OM microtome (Microm International) and transferred onto Superfrost Plus glass slides.

For GFAP staining, cryosections were washed with PB prior to the 1 h-blockage with 2% BSA supplemented with 0.2% CWFG and 0.1% Triton. After the blockage, sections were washed (3×, 5 min each) and incubated overnight with the primary polyclonal chicken anti‐GFAP antibody (LSBio, Seattle, USA) (1:1000 in 1:10 blockage buffer) at 4 °C. Then, they were washed (3×, 5 min each) and incubated for 1 h with the Alexa 488 anti‐chicken secondary antibody (1:1000 in 1:10 blockage buffer) (Abcam, Cambridge, UK). Finally, the sections were washed again and rinsed with ultrapure water prior to mounting with Mowiol 4–88 supplemented with DAPI (0.15 µg/ml).

For IBA-1 staining, cryosections were washed with PB prior to 1 h-blockage with 2% BSA. After the blockage, sections were washed (3×, 5 min each) and incubated overnight with the primary polyclonal rabbit anti‐IBA-1 antibody (FUJIFILM Wako Pure Chemical Corporation, Osaka, Japan) (1:1000 in 1:10 blockage buffer) at 4 °C. Then they were washed (3×, 5 min each) and incubated for 1 h with the anti‐rabbit secondary antibody Alexa 488 (1:1000 in 1:10 blockage buffer) (Invitrogen, Thermo Fisher Scientific, Waltham, USA). Afterward, sections were washed again and rinsed with ultrapure water prior to mounting with Mowiol 4–88 supplemented with DAPI (0.15 µg/ml).

For F4/80 staining, cryosections were washed with PB prior to fixation with 4% PFA for 8 min and washed again (2×, 5 min each) with PB before 1 h-blockage with 1% non-fat dry milk (Blotto) supplemented with 0.1% Tween. After the blockage, sections were washed (3×, 5 min each) and incubated overnight with the primary polyclonal rat anti‐F4/80 antibody (Acris Antibodies GmbH, Herford, Germany) (1:400 in 1:10 2% BSA supplemented with 0.1% Triton) at 4 °C. Then they were washed (3×, 5 min each) and incubated for 1 h with the biotinylated anti‐rat secondary antibody (1:500 in 1:10 blockage buffer) (Vector Laboratories, Burlingame, USA). Before and after the incubation with streptavidin Alexa 488 (1:1000 in 1:10 blockage buffer) (Invitrogen, Thermo Fisher Scientific, Waltham, USA) for 1 h, sections were washed again and rinsed with ultrapure water prior to mounting with Mowiol 4–88 supplemented with DAPI (0.15 µg/ml).

For CD31 staining, cryosections were washed with PB prior to 1 h-blockage with 2% BSA supplemented with 0.2% cold water fish gelatin (CWFG) and 0.1% Triton. Sections were then washed with phosphate buffer (3×, 5 min each) and incubated overnight with the primary polyclonal goat anti‐CD31 antibody (R&D systems, Minneapolis, USA) (1:100 in 1:10 blockage buffer) at 4 °C. Then, they were washed again with phosphate buffer (3×, 5 min each) and incubated for 1 h with the biotinylated anti‐goat secondary antibody (1:500 in 1:10 blockage buffer) (Vector Laboratories, Burlingame, USA). Before and after the incubation with streptavidin Alexa 488 (1:1000 in 1:10 blockage buffer) (Invitrogen, Thermo Fisher Scientific, Waltham, USA) for 1 h, sections were washed again and rinsed with ultrapure water prior to mounting with Mowiol 4–88 (Carl Roth, Karlsruhe, Germany) supplemented with DAPI (0.15 µg/ml).

All images were generated using an Axio Imager fluorescence microscope with a Plan-Neofluar 40 × /1.30 Oil M27 objective (Carl Zeiss AG) and Zen Software (Carl Zeiss Microscopy). For the quantitative evaluation of immunofluorescence in the posterior eye section, the retinal area was gated, and the integrated fluorescence density of each gated area was quantified using ImageJ version 1.52.

### RNA analysis

Total RNA samples from retinas were extracted using TRIzol (Invitrogen, Waltham, USA) following the manufacturer’s instructions for RNA isolation. After determining the RNA concentration using a NanoDrop spectrophotometer (Thermo Fisher Scientific, Waltham, USA), equal amounts of total RNA were reversed transcribed into first-strand cDNA using iScript cDNA Synthesis Kit (Bio-Rad, Hercules, USA) according to manufacturers’ instructions. Quantitative real-time RT-PCR analyses were performed using the iQ5 Real-Time PCR Detection System (Bio-Rad). RNA that was not reverse transcribed served as negative control for real-time RT-PCR, and for relative quantification, GNB2L was used. Quantification and analysis were performed using BioRad iQ5 software (BioRad), and data was processed using Microsoft Excel 2019 and GraphPad Prism 6.0. The primer sequences (Invitrogen, Waltham, USA) used were as follows:

- 5′-TCTGCAAGTACACGGTCCAG-3′; GNB2L forward
- 5′-ACGATGATAGGGTTGCTGCT-3′; GNB2L reverse
- 5′-CTCTCTAAGGCTACAGGCTGCT-3′; Cd68 forward
- 5′-TCACGGTTGCAAGAGAAACA-3′; Cd68 reverse
- 5′-CATCCACGTGTTGGCTCA-3′; Ccl2 forward
- 5′-GATCATCTTGCTGGTGAATGAGT-3′; Ccl2 reverse
- 5′-GGATTTGCAGGGAGGAAAA-3′; IBA-1 forward
- 5′-TGGGATCATCGAGGAATTG-3′; IBA-1 reverse
- 5′-TCGAGATCGCCACCTACAG-3′; GFAP forward
- 5′-GTCTGTACAGGAATGGTGATGC-3′; GFAP reverse
- 5′-GGGCTGTCACGGAGATCA-3′; iNOS forward
- 5′-CCATGATGGTCACATTCTGC-3′; iNOS reverse
- 5′-AGTTGACCGACCCCAAAAG-3′; Il-1beta forward
- 5′-AGCTGCATGCTCTGATCAGG-3′; Il-1beta reverse
- 5′-GCTACCAAACTGGATATAATCAGGA-3′; Il-6 forward
- 5′-CCAGGTAGCTATGGTACTCCAGAA-3′; Il-6 reverse
- 5′-TCTTCTCATTCCTGCTTGTGG-3′; TNF-alpha forward
- 5′-GGTCTGGGCCATAGAACTGA-3′; TNF-alpha reverse

### Statistical analysis

Data are expressed as mean ± SD. Statistical evaluation was performed using GraphPad Prism Software 6.0. Student’s *t*-test for unpaired data, one-way ANOVA, and two‐way ANOVA with a Sidak’s or Tukey’s multiple comparison test were employed to evaluate statistical significance. Significant differences were indicated as *(*P* < 0.05), **(*P* < 0.01), *** (*P* < 0.001), and **** (*P* < 0.0001) related to control unless otherwise stated.

## Data Availability

All data needed to evaluate the conclusions in the paper are present in the paper. Additional data related to this paper may be requested from the authors.
